# Analgesic Effect of Passive Range-of-Motion Exercise on the Healthy Side for Pain after Total Knee Arthroplasty: A Prospective Randomized Trial

**DOI:** 10.1155/2023/1613116

**Published:** 2023-06-14

**Authors:** Shuichi Eto, Motoki Sonohata, Yasuo Takei, Masaya Ueno, Norio Fukumori, Masaaki Mawatari

**Affiliations:** ^1^Department of Orthopaedic Surgery, Faculty of Medicine, Saga University, 5-1-1 Nabeshima, Saga 849-8501, Japan; ^2^Center for Advanced Comprehensive Recovery, Saga University Hospital, 5-1-1 Nabeshima, Saga 849-8501, Japan; ^3^Education and Research Center for Community Medicine, Faculty of Medicine, Saga University, 5-1-1 Nabeshima, Saga 849-8501, Japan

## Abstract

**Background:**

Exercise can reduce the pain threshold momentarily and induce analgesia, which is called exercise-induced hypoalgesia (EIH). Exercise therapy for inducing EIH may be an effective treatment option for pain. We aimed at investigating whether continuous passive motion (CPM) on both healthy and affected sides could induce EIH and reduce pain in the operated knee in patients after unilateral total knee arthroplasty (TKA). *Patients and Methods*. In this prospective randomized controlled trial, participants were randomly assigned to two groups: a bilateral group that received bilateral exercise on the operated and healthy sides and a unilateral group that received exercise therapy only on the affected side. We enrolled 40 patients aged ≥60 years who were scheduled to undergo unilateral TKA. Visual analogue scale (VAS) scores and range of motion (ROM) on the operated side were measured immediately before and after CPM on postoperative days 2, 4, 7, and 14. The primary outcome was the difference in the VAS scores before and after CPM on postoperative day 14. The secondary outcome was the difference in the ROM before and after CPM on postoperative day 14.

**Results:**

Comparison of VAS scores before and after CPM showed no significant intergroup differences on all measurement dates. However, there was a significant difference in values on day 14 (*P* < 0.05). Both groups showed an increase in ROM after CPM, with significant increments observed on days 2 and 4 in the bilateral group and on day 14 in the unilateral group. There was no significant difference in values on postoperative day 14.

**Conclusion:**

Post-TKA pain was reduced by performing the same exercise on the healthy knee during CPM therapy. This could be due to EIH, and the results indicated that EIH can also influence postoperative pain immediately after surgery.

## 1. Introduction

Total knee arthroplasty (TKA), which is an effective therapeutic option for knee osteoarthritis, has yielded good outcomes [1]. However, it is associated with severe postoperative pain: 60% of patients who undergo TKA experience severe pain and 30% experience moderate pain [2, 3]. Some patients may avoid the surgery itself due to the pain associated with the surgical procedure [4], thus preventing improvement in their knee function. In addition, postoperative pain can affect functional improvements, such as postoperative range-of-motion (ROM) improvements, making pain control even more important. Although pain control is mainly achieved with medications, opioid usage should preferably be minimized [4, 5], and methods to decrease pain without relying on medications are desirable in this regard. Continuous passive motion (CPM) is a widely used postoperative exercise therapy that is expected to improve soft tissue repair and function and shorten hospital stay [6]. However, some studies yielded negative findings for its effectiveness, and guidelines have made insufficient recommendations [6–8]. The reason for this may be the severe pain associated with exercise therapy, which is initiated in the acute postoperative period.

Exercise can effectively sustain a healthy and fulfilling life in healthy individuals and in patients with pain [9–11]. Physical activity and regular exercise are guideline-recommended treatments for chronic pain [10] that positively influence physical and mental health, including maintaining and improving cardiovascular function, stress, mood, sleep, and sexual health [11]. Exercise also reduces the pain threshold for a certain period of time and induces analgesia, which is called exercise-induced hypoalgesia (EIH) [12, 13]. Although EIH is occasionally impaired in patients with chronic pain such as fibromyalgia, induction of EIH through exercise in pain-free areas can reduce the pain threshold even in these patients [14, 15]. The effectiveness of exercise therapy may be further improved by inducing EIH. However, to our knowledge, no previous study has attempted to validate EIH in the acute postoperative period.

We hypothesized that simultaneous exercise of other parts of the body during CPM could induce EIH and alleviate pain in the operated knee. Thus, we aimed at investigating whether CPM on the healthy and affected sides could induce EIH and reduce pain in the operated knee after unilateral TKA.

## 2. Materials and Methods

The study was conducted at a university hospital in Japan from November 2019 to October 2021. The protocol of this trial was approved by the institutional review board and registered with the University Hospital Medical Information Network Clinical Trials Registry (UMIN000045437, https://center6.umin.ac.jp/cgi-open-bin/ctr_e/ctr_view.cgi?recptno=R000048173). The study was conducted in accordance with the Declaration of Helsinki. All patients provided written informed consent.

### 2.1. Participants

We enrolled patients aged ≥60 years who were scheduled for unilateral TKA at the university hospital. This age group was preferred because the principal indication for TKA is ≥60 years at our institution. Patients were excluded based on the following criteria: severe pain or deformity of the knee on the contralateral side, chronic opioid use, diabetes mellitus due to the possibility of peripheral neuropathy, rheumatoid arthritis, necessity of anticoagulants, or impairment of cognitive function.

### 2.2. Randomization and Blinding

This study was conducted as a prospective randomized controlled trial. Participants were randomly assigned to two groups: a bilateral group that performed bilateral exercise on the affected and healthy sides and a unilateral group that performed exercise therapy only on the affected side. Computer-generated block randomization (block size, 4, revealed to the study group at the first exercise) was used for randomization such that both groups contained equal numbers of patients. Group allocation was performed by the first author; the examiner and patients were blinded to group assignment until the CPM practice began. As the groups performed different CPM practices, the participants could not be masked to the group assignment.

### 2.3. Interventions

#### 2.3.1. Surgery

A standard TKA was performed under spinal anesthesia via a medial parapatellar approach with an air tourniquet. The decision for spinal anesthesia was determined by the anesthesiology department. Five expert surgeons performed the procedures using identical protocols and selected the appropriate model according to the patient's bone quality and size. We utilized the following four medical implants: Persona (Zimmer Biomet, Warsaw, IN), Trimax (Ortho Development, Draper, UT), Triathlon (Stryker, Kalamazoo, MI), and Initia (KYOCERA, Osaka, Japan). An intraoperative periarticular multimodal drug cocktail injection (levobupivacaine hydrochloride, 50 mg, and triamcinolone acetonide, 40 mg) was administered, and a suction drain was placed in the joint cavity. The suction drain was removed on postoperative day 2.

#### 2.3.2. Postoperative Analgesia

Flurbiprofen axetil (50 mg) was mixed in the infusion (500 mL), and five infusions containing 250 mg of flurbiprofen axetil were administered over the next morning. On the evening of the day of surgery, celecoxib administration (400 mg/day for 2 weeks) was started. As rescue pain medications, diclofenac sodium (suppository), tramadol hydrochloride (100 mg, intramuscular injection), and pentazocine (15 mg, intramuscular injection) were administered according to the patient's condition. The dosage was appropriately adjusted based on age, weight, and renal function. We excluded patients who used analgesics within 3 h of beginning the CPM exercise because their effects were rather obvious.

#### 2.3.3. CPM and Rehabilitation

Automatic knee joint movement was started on the day after surgery depending on pain, and CPM was started on postoperative day 2. The following instruments were used: ARTROMOT-K1 (Medireha GmbH, Umkirch, Germany) and Spectra (Kinetec Medical Products, Aldershot, UK). CPM was performed once a day for 20 min each time. The setting angle was determined by flexing the knee before the start of CPM therapy and gradually increasing the angle depending on the patient's pain tolerance. Other postoperative rehabilitation procedures were performed according to the same protocol for both groups. The load was increased as the patients' pain and motor function improved.

### 2.4. Measurements

The following patient information was collected preoperatively: age, sex, body mass index (BMI), diagnosis, visual analogue scale (VAS) score, knee joint ROM, and Kellgren–Lawrence classification for the operated and nonoperated sides. For postoperative evaluation, the time from return to first use of rescue analgesia and the total amount of rescue analgesia during the study period were also measured. VAS scores and ROM on the operated side were measured immediately before and after CPM on postoperative days 2, 4, 7, and 14. The 10 m walk test was performed on postoperative days 7 and 14 to evaluate walking ability. The primary outcome was the difference in the VAS scores before and after CPM on postoperative day 14. This primary outcome was selected based on the expectation that the impact of nociceptive pain associated with surgery decreases in 2 weeks. The secondary outcome was the difference in the ROM before and after CPM and the 10 m walk test result on postoperative day 14.

#### 2.4.1. VAS Score

Pain severity was measured using the VAS. Patients were instructed to mark their pain before and after CPM on a 100 mm horizontal line where the most severe pain was noted on the right end and no pain was noted on the left end [16]. Results were presented in mm.

#### 2.4.2. ROM

ROM was measured using a goniometer with a 30 cm movable arm in the supine position. The goniometer was centered over the joint, with one arm directed along the fibula to the external condyle and the other along the femur to the greater trochanter [17]. Each patient demonstrated flexion and extension tolerance before CPM, and the angles were measured. Immediately after CPM, the angle was measured again.

#### 2.4.3. 10 m Walk Test

The 10 m walk test was conducted to evaluate postoperative walking ability [18]. Patients were instructed to walk at a comfortable speed on a 10-meter walking path from a static position using a cane, and the time was recorded.

### 2.5. Sample Size Calculation

In an interim analysis of 26 patients (unpublished data), the mean and standard deviation (SD) values of the primary outcome were −11.7 (SD, 14.2) and 1.7 (SD, 10.6) in the bilateral and unilateral groups, respectively. Based on these data and by assuming a population SD of 10, statistical power of 0.8, and significance level of 0.05, a sample size of 32 patients (both groups combined) was required. To account for possible attrition, we set the target overall caseload at 40 cases (20 in each group). Block randomization and sample size calculations were performed under the direction of a statistician who was not involved in the trial.

### 2.6. Statistical Methods

We performed an intention-to-treat analysis, incorporating all randomized participants. Statistical analyses were conducted using JMP Pro version 15.2.0 (SAS Institute Inc., Cary, NC, USA). An unpaired *t*-test was used for age, and the Mann–Whitney *U* test was used for BMI, time from return to first use of rescue analgesia, total amount of rescue analgesia, VAS score, ROM, and 10 m test. Categorical data (e.g., sex, diagnosis, implant, and Kellgren–Lawrence classification) were compared using chi-square and Fisher's exact tests. A *P* value <0.05 was considered statistically significant.

## 3. Results

### 3.1. Patient Characteristics

**Figure 1:**
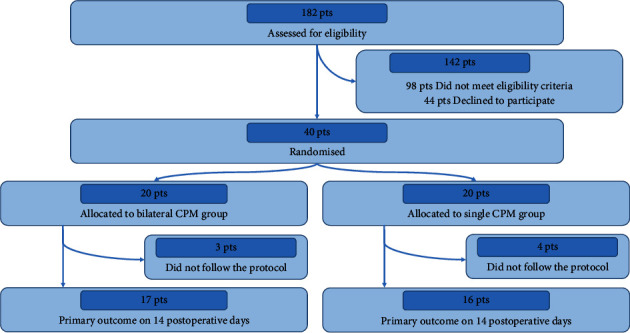
Flowchart of participant selection.

Figure 1 outlines the flowchart for participant selection in the study. Overall, 182 patients underwent TKA between November 2019 and October 2021 and were screened for eligibility. Most screened patients (*n* = 98, 53.8%) were not eligible due to complications. In total, 40 eligible participants who agreed to provide informed consent were randomly allocated to two groups. According to the protocol, patients who could not be prescribed nonsteroidal anti-inflammatory drugs postoperatively owing to chronic kidney disease were excluded, resulting in 17 patients in the bilateral group and 16 in the unilateral group. The baseline characteristics of the patients in both groups (20 patients in each group) are shown in Table 1. The two groups showed no significant differences in preoperative baseline characteristics (Table 1). No patient required rescue analgesia within 3 h of beginning CPM.

### 3.2. Postoperative Rescue Analgesia

The median time from return to first use of rescue analgesia was 442.5 min (270, 822.8) in the bilateral group and 401.5 min (143.8, 1031.3) in the unilateral group, with no significant difference between the groups (*P*=0.85). There was also no significant between-group difference in the total amount of diclofenac sodium used (150 mg (87.5, 362.5) in the bilateral group and 175 mg (100, 375) in the unilateral group, *P*=0.76). Both tramadol hydrochloride and pentazocine were used only on the day of surgery. Four patients in the bilateral group used tramadol hydrochloride once, six in the unilateral group used tramadol hydrochloride once, and one in the unilateral group used tramadol hydrochloride twice, with no significant difference in dosage (*P*=0.204). Pentazocine was used by one patient in the unilateral group (single use) but not used in the bilateral group, with no significant between-group difference (*P*=0.15).

### 3.3. VAS Scores

**Figure 2:**
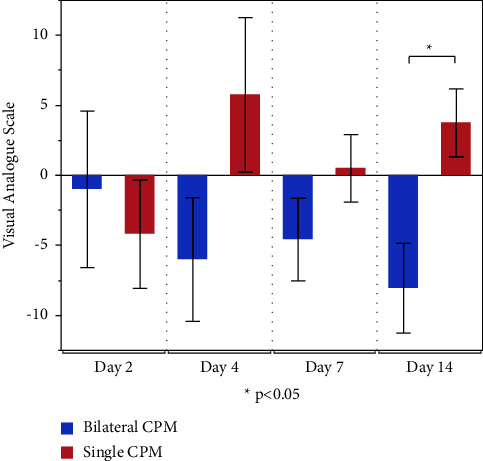
Difference between before and after CPM for visual analogue scale in the intention-to-treat analysis. The data bar shows mean ± standard error (SE).

Table 2 shows the VAS scores before and after CPM with the intention-to-treat analysis. Comparison of VAS scores before and after CPM showed no significant differences on all measurement dates in both groups. However, the scores in the unilateral group increased after CPM on all days except for day 2, whereas scores in the bilateral group decreased after CPM, as shown in the graph of the difference between the before and after CPM values (Figure 2). The data on differences in values suggested that pain decreased after CPM administration in both groups on day 2, with no significant difference; after day 4, while the pain decreased after CPM in the bilateral group, it increased in the unilateral group, with a significant difference between the two groups on postoperative day 14. The mean difference between groups was 9.5 mm (95% confidence interval 2.26 to 16.74), significantly lower in the bilateral CPM group (*P*=0.03). These results were similar in the per-protocol analysis (11.81 mm (3.54 to 20.08), *P*=0.01).

### 3.4. ROM

**Figure 3:**
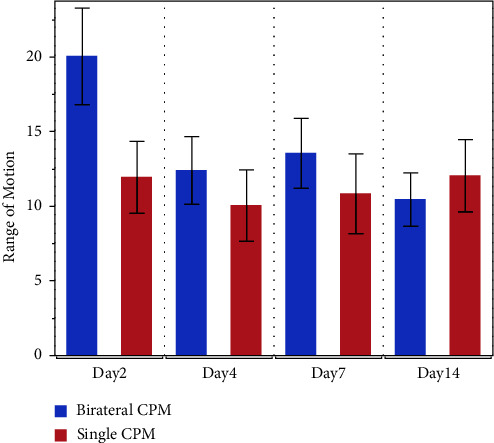
Difference between before and after CPM for range of motion in the intention-to-treat analysis. The data bar shows mean ± standard error (SE).

Both groups showed an increase in ROM after CPM, with significant increments observed on postoperative days 2 and 4 in the bilateral group and on postoperative day 14 in the unilateral group (Table 3).  Figure 3 presents the difference in values before and after CPM. Both groups showed an increase over time, with no significant difference between the two groups. On postoperative day 14, mean difference between groups was 1.6° (95% confidence interval −4.49 to 7.69), with no significant difference (*P*=0.87). These results were similar in the per-protocol analysis (1.98° (−5.11 to 9.07), *P*=0.81).

### 3.5. 10 m Test

The 10  m test results for the bilateral and unilateral groups were 16.3 s (14.43, 18.18) and 14.7 s (13.12, 16.68) on postoperative day 7 and 15 s (12.88, 17.63) and 13.6 s (12.4, 14.63) on postoperative day 14. The two groups showed no significant difference (*P* > 0.05). These results were similar in the per-protocol analysis. On postoperative day 14, the mean between-group difference was −1.54 s (95% confidence interval −3.39 to 0.3), which was not significant (*P*=0.11).

## 4. Discussion

In this study, there was a significant difference in the primary outcome between the two groups, i.e., the difference in VAS scores before and after CPM on postoperative day 14, with a decrease in the bilateral group and an increase in the unilateral group. The secondary outcome, i.e., the difference in ROM before and after CPM on postoperative day 14, and the 10 m test results were not significantly different between the groups. Simultaneous exercise of the healthy side during rehabilitation with CPM was indicated to decrease pain during exercise on the operated side, while no significant improvement in motor function was observed.

The number of patients undergoing TKA has been steadily increasing, with a projected 3.48 million patients expected to have undergone the procedure in the United States by 2030 [19]. While the number of surgeries is increasing, 20% of patients who undergo primary TKA experience long-lasting pain and are not satisfied with the results [3]. Factors contributing to this dissatisfaction include patient expectations before surgery, the degree of improvement in knee function, and pain relief after surgery [20]. Postoperative rehabilitation is important for pain relief and functional recovery, but the rehabilitation protocols vary by country and region [6]. CPM is a physical therapeutic modality wherein the knee is flexed and extended with electric equipment in an altruistic manner and is performed similarly in many countries. We investigated a new analgesic method using modification of CPM during postoperative exercise therapy; this is the first study to determine whether EIH influences postoperative pain in the acute phase. In addition, while automatic exercise (isometric and aerobic exercise) has been used to validate EIH [21–23], this study used instrumented passive, dynamic, and nonresistant exercise, which is different from that of the previous studies. The exercise site was chosen to be the healthy knee in anticipation of early functional recovery through activation of deep sensory perception in the operated knee joint by mirroring of the healthy knee exercise [24]. CPM is a passive exercise; thus, analgesia through attentional dispersion can also be expected [25].

Postoperative CPM was thought to increase pain, which decreased after the first CPM on postoperative day 2 in both groups. Loading and repetitions performed for contraction failure increased postoperative knee pain, although this increase was transient [26]. Knee pain increased during exercise, while the increased pain resolved with cessation; thus, no significant difference in pain was observed before and after CPM. The unilateral group showed an increase in pain after CPM following postoperative day 4. The subjective perception of pain increases with continued unpleasant stimuli, i.e., temporal summation of pain (TSP) [12, 21], and repeated exercise of the painful area after TKA may have caused TSP, resulting in increased pain after CPM. Exercise has been reported to decrease TSP [12] only when it is performed outside the pain site. In contrast, exercise at the pain site does not cause EIH and does not decrease TSP [14]. However, the post-CPM pain was reduced when the healthy side was also exercised. Thus, simultaneous exercise of the healthy side could have induced EIH and reduced pain. The results also suggest that the pain-relieving effect could be enhanced by repeated exercise on the healthy knee over a short period of time. This may be due to the potentiating effect of EIH, wherein exercise reduced pain and changed the appraisal of nociceptive stimuli [27, 28]. No difference was observed in ROM or 10 m test results between the two groups, suggesting that analgesia did not improve functional aspects of the patients. Like previous studies, the effect of CPM itself was limited to the acute postoperative period [29]. The experience and education of pain relief from exercise, however, may promote habitual exercise, which could contribute to functional improvement [28].

The limitations of this study include the unblinded design, the small sample size, and the fact that no confirmatory analyses could be performed to determine whether the effects were truly due to EIH. This study could not be performed with blinding, and patients become aware of their group at the time of CPM. This inability to blind may have affected the outcome. Although the sample size was small, we believe our results are reliable because we used statistically evaluated values. Although the effects of EIH could not be confirmed because we did not measure thresholds, tolerance, and/or rating of noxious stimuli, exercise for the healthy knee reduced pain during CPM. These findings represent a novel aspect of exercise therapy in the acute postoperative period. However, additional research is needed, and novel rehabilitation methods are expected to be developed based on the results of this study.

## 5. Conclusions

Post-TKA pain was reduced by performing the same exercise on the healthy knee during CPM therapy. This effect could be attributed to EIH, and the results indicated that EIH is also effective against acute postoperative pain. Although further research is needed, these new findings are expected to be useful for the future development of rehabilitation methods.

## Figures and Tables

**Table 1:** Baseline characteristics of the patients.

	Bilateral group (*N* = 20)	Unilateral group (*N* = 20)	*P* value
Age (yrs.)	73.8 ± 6.6	74.7 ± 5.6	0.84
Sex	Male, 6; female, 14	Male, 4; female, 16	0.72
BMI (kg/m^2^)	25.24 (23.31, 28.52)	25.28 (23.87, 27.98)	0.88
Visual analogue scale (mm)	49 (11.25, 62.75)	53.5 (19.25, 71.5)	0.42
Range of motion (°)	110 (100, 128.75)	110 (100, 118.75)	0.67
Implant (P; T; Tr; I)	10; 3; 4; 3	14; 4; 1; 1	0.113

*Diagnosis of total knee arthroplasty*			
Osteoarthritis	20 (100%)	17 (85%)	0.072
Osteonecrosis	0 (0%)	3 (15%)	

*Kellgren–Lawrence classification (operated side) (n (%))*			
0	0 (0%)	0 (0%)	0.95
1	0 (0%)	0 (0%)	
2	1 (5%)	1 (5%)	
3	11 (55%)	10 (50%)	
4	8 (40%)	9 (45%)	

*Kellgren–Lawrence classification (nonoperated side) (n (%))*			
0	0 (0%)	0 (0%)	0.32
1	1 (5%)	3 (15%)	
2	5 (20%)	8 (40%)	
3	7 (35%)	3 (15%)	
4	1 (5%)	3 (15%)	
Post-total knee arthroplasty	6 (30%)	3 (15%)	

P, Persona; T, Trimax; Tr, Triathlon; I, Initia.

**Table 2:** Visual analogue scale before and after CPM.

Day	Bilateral group			Unilateral group		
	Before CPM	After CPM	*P* value	Before CPM	After CPM	*P* value
2	46.8 (27.5, 58)	46.1 (24.3, 63.8)	0.88	44.6 (27.3, 66.5)	38.6 (23.5, 54.8)	0.41
4	41.5 (24.3, 58)	36.1 (25.3, 43.5)	0.27	32.6 (17.5, 48)	35.3 (17.8, 53.8)	0.85
7	41.1 (23, 56.8)	37.3 (20.5, 55.8)	0.53	26.1 (7.8, 40)	30.2 (8.3, 42.8)	0.53
14	32.2 (13.3, 41.3)	24.7 (4.2, 41)	0.28	28.9 (16.5, 41.8)	30.9 (14.8, 38.8)	0.86

*P* value; before CPM vs after CPM.

**Table 3:** Range of motion before and after CPM.

Day	Bilateral group			Unilateral group		
	Before CPM	After CPM	*P* value	Before CPM	After CPM	*P* value
2	47.9 (30, 58.8)	67.9 (50, 75)	0.003^*∗*^	54.6 (36.3, 63.8)	66.5 (46.3, 90)	0.12
4	62.5 (50, 75)	74.9 (61.3, 85)	0.035^*∗*^	74.4 (55, 91.5)	84.4 (67.5, 100)	0.13
7	73.5 (60, 85.8)	87.6 (70, 103.8)	0.054	85.3 (65, 105)	96.1 (81.3, 110.8)	0.14
14	85.9 (66.3, 104)	96.3 (81.8, 110.8)	0.081	88.2 (80, 100)	100.3 (90, 110)	0.048^*∗*^

*P* value; before CPM vs after CPM.

## Data Availability

The Excel data used to support the findings of this study are available from the corresponding author upon request.
